# Adaptation and validation of the Greek version of the Communication and Language Assessment questionnaire for persons with Multiple Sclerosis (CLAMS)

**DOI:** 10.1093/arclin/acae015

**Published:** 2024-03-09

**Authors:** Nefeli Dimitriou, Grigorios Nasios, Anastasia Nousia, Emmanouil Anyfantis, Lambros Messinis, Georgios Dimakopoulos, Sarah El-Wahsh, Christos Bakirtzis, Vasiliki Kostadima, Spiridon Konitsiotis

**Affiliations:** Department of Speech and Language Therapy, University of Ioannina, Ioannina, Greece; Department of Speech and Language Therapy, University of Ioannina, Ioannina, Greece; Department of Speech and Language Therapy, University of Peloponnese, Kalamata 24100, Greece; Department of Speech and Language Therapy, University of Ioannina, Ioannina, Greece; Lab of Cognitive Neuroscience, School of Psychology, Aristotle University of Thessaloniki, Thessaloniki, Greece; BIOSTATS, Science and Technology Park of Epirus, Ioannina, Greece; Discipline of Speech Pathology, Faculty of Medicine and Health, The University of Sydney, Camperdown, NSW, Australia; B’ Department of Neurology, AHEPA University Hospital, Aristotle University of Thessaloniki, Thessaloniki, Greece; Department of Neurology, University of Ioannina Medical School, Ioannina, Greece; Department of Neurology, University of Ioannina Medical School, Ioannina, Greece

**Keywords:** Multiple sclerosis, Validation, Patient reported outcome measure, Communication, Language

## Abstract

**Objective:**

The aim of the present study was to validate the Communication and Language Assessment questionnaire for persons with Multiple Sclerosis (CLAMS) into the Greek language.

**Method:**

106 Persons with Multiple Sclerosis (PwMS) and 51 healthy controls (HCs) participated in this study. We evaluated patients’ cognitive abilities with the Brief International Cognitive Assessment for Multiple Sclerosis (BICAMS). All PwMS completed the CLAMS and three additional questionnaires (Speech Pathology-Specific Questionnaire for persons with Multiple Sclerosis, SMS; Stroke and Aphasia Quality of Life Scale-39, SAQOL-39; the Beck Depression Inventory Fast Screen, BDI-FS), and all HCs filled in the CLAMS.

**Results:**

The internal consistency of the CLAMS was excellent (*a* = 0.933) for the PwMS and a significant difference was found between PwMS and HCs for the total CLAMS score. Statistical analyses showed a significant positive correlation between the CLAMS and the other questionnaires (SMS, BDI, and SAQOL-39) and a statistically significant negative correlation between the CLAMS and the three subtests of the BICAMS (Symbol Digit Modalities Test, Greek Verbal Learning Test-II, and Brief Visuospatial Memory Test-Revised). There was no correlation between the CLAMS and participants’ age, disease duration, and disease type.

**Conclusion:**

The Greek version of the CLAMS is a valid self-reported questionnaire for the evaluation of language and communication symptoms in PwMS.

## Introduction

Multiple sclerosis (MS) is a chronic, unpredictable, disabling disease of the central nervous system in which inflammation, demyelination, and axonal loss occurs (Oh et al., 2018). There are three main types of MS: relapsing-remitting MS, primary progressive MS, and secondary progressive MS (Dobson & Giovannoni, 2019). The classification of disease type depends on its activity and progression. Its course can vary across individuals, leading to a constellation of different symptoms including motor, sensory, cognitive, speech, language and/or mood changes (Svindt et al., 2020).

Specifically, MS negatively affects several aspects of cognitive function including attention, information processing, memory, learning (Dardiotis et al., 2018), executive function (Clough et al., 2018), and visuospatial abilities (Van Der Ham et al., 2022). These symptoms can significantly affect a person’s quality of life, employment status, daily functioning, independence, and participation in social activities (Pérez-Martín et al., 2017; Svindt et al., 2020). In addition to cognitive symptoms, difficulties with communication, such as language and speech, are also observed in approximately one third of Persons with Multiple Sclerosis (PwMS) (Johansson et al., 2021).

Furthermore, recent studies highlight speech disorders and subsequent limited participation resulting from MS (Feenaughty, 2023). The most common of these problems are dysarthria and dysphonia (Renauld et al., 2016; Rusz et al., 2019; Yorkston et al., 2003). Specifically, the demyelinating lesions caused by MS may result in spasticity, weakness, slowness, and/or ataxic in coordination of the lips, tongue, mandible, soft palate, vocal cords, and diaphragm. Therefore, articulation, speaking rate, intelligibility, and natural flow of speech in conversation are the area’s most commonly affected in PwMS (Murdoch & Theodoros, 2000). Moreover, abnormalities are observed in various language and cognitive aspects in PwMS. Specifically word finding difficulty, paraphasias, poor topic maintenance, difficulty with discourse production, reduced cognitive flexibility, slowed information processing speed and attestation, and reduced language comprehension (most often with syntactically complex sentence structures or non-literal utterances such as metaphors, idioms, and ambiguous language) (El-Wahsh et al., 2022; Renauld et al., 2016). Evidence from Greek-speaking PwMS has shown difficulty with retrieval of nouns and verbs (Kambanaros et al., 2017), as well as complex sentence processing deficits (Fyndanis et al., 2020).

Communication changes observed in PwMS have been shown to negatively affect ability to participate in effective and efficient conversations (Arrondo et al., 2010; Carotenuto et al., 2018; El-Wahsh et al., 2020a; Grossman et al., 2009; Johansson et al., 2021; Lethlean & Murdoch, 1994; Renauld et al., 2016). Reduced quality of life and participation and activity restrictions because of communication changes can result in emotional distress, anxiety, feelings of incompetence, and embarrassment (Klugman & Ross, 2002). Thus, clinicians should be mindful of the possible negative impacts of communication changes and lower speech usage levels on patients’ functional communication and use a variety of measures to capture these communication changes (El-Wahsh et al., 2022; Feenaughty et al., 2018; Yorkston et al., 2014).

A speech language pathology evaluation concerning possible communication changes is crucial for a valid assessment and appropriate management based on each individual’s needs. The Communication and Language Assessment questionnaire for persons with Multiple Sclerosis (CLAMS) is a patient-reported outcome measure (PROM) developed specifically for PwMS to evaluate communication symptoms (El-Wahsh et al., 2020b). This tool focuses on self-perceived communication ability. As a PROM, this tool can help clinicians with symptom evaluation, progression and monitoring of symptoms, and to guide treatment (Noffs et al., 2018). The aim of the present study was to adapt and validate the CLAMS into the Greek language and to investigate its psychometric properties.

## Materials and methods

### Participants

A total of 106 PwMS participated in this study from August 2021 to October 2022. The healthy control (HC) group comprised of 51 age, sex, and education level matched individuals. Patients were recruited from the Neurologic Clinic of University Hospital of Ioannina and a private medical center in Ioannina, Greece. Participants were included in the study if they met the following criteria: (1) diagnosis of MS according to the McDonald revised 2017 criteria, (2) ≥18 years of age, and (3) stable disease defined as the absence of clinical and radiological activity or disease progression over the last 6 months prior to participation in the study. Exclusion criteria included the presence of psychiatric disorder (e.g., untreated depression, psychotic state, and alcohol or illegal drug abuse), other neurological disorder/s (e.g., epilepsy, stroke, Parkinson’s disease, and traumatic brain injury) and visual/hearing impairment.

### Cognitive assessment

Cognitive function in PwMS was evaluated with the Greek version of the Brief International Cognitive Assessment for Multiple Sclerosis (BICAMS; Polychroniadou et al., 2016), which includes (1) the Symbol Digit Modalities Test (SDMT; Smith, 1982) to assess information processing speed, (2) the Greek Verbal Learning Test (GVLT; Vlahou et al., 2013) to assess verbal memory, and (3) the Brief Visuospatial Memory Test-Revised (BVMT-R) to assess visual/spatial memory (Benedict et al., 1996).

### CLAMS questionnaire

The CLAMS consists of 11-items and each item is graded on a 4-point Likert scale (0 “never” to 3 “usually or always”). The maximum total score is 33. The original version of the tool (El-Wahsh et al., 2020b) was translated into Greek by three natural Greek-speakers, including two speech language pathologists and a neurologist. A clinical neuropsychologist performed the back translation. No items from the original version of the CLAMS were omitted/changed or replaced. This final Greek version was trialed with nine control participants to establish its perceptiveness and was approved by the research team.

### Other PROMs

In addition to the Greek version of the CLAMS, all participants completed three additional PROMs. The purpose of using the tools listed below [Speech Pathology-Specific Questionnaire for persons with Multiple Sclerosis (SMS), Stroke and Aphasia Quality of Life Scale-39 (SAQOL-39), and Beck Depression Inventory Fast Screen (BDI-FS)] was to evaluate the criterion validity (i.e., the extent to which a tool agrees with another tool evaluating a similar construct).

1. The SMS ( El-Wahsh et al., 2019) Greek version (Dimitriou et al., 2023) includes 16 questions to assess speech/voice, language, and swallowing abilities in PwMS. Each item is graded on a five-point Likert scale (0 “never” to 4 “always”). The maximum total score is 64.
2. The SAQOL-39 (Hilari et al., 2003) Greek version (Kartsona & Hilari, 2007) is a 39-item PROM that has been developed to assess health-related quality of life in people with chronic aphasia. The tool addresses four key domains, including physical, psychosocial, communication, and energy. There are two possible answers for each item (yes or no). A higher score indicates greater severity.
3. The short form of the BDI-FS (Benedict et al., 2003) Greek version (Beck et al., 2003) is a reliable and quick screening instrument with seven-items (each with four possible answers) to assess depression. MS is a chronic progressive neurological disease with psychological and psychiatric implications, and accordingly, this tool was included to help capture the person’s mood (Bakirtzis et al., 2022).

Finally, we used the 36-Item Short Form Health Survey questionnaire (SF-36; Ware et al., 1993), Greek version (Pappa et al., 2005) to collect general information regarding the person’s quality of life.

### Procedure

Both PwMS and HCs completed all tasks in a quiet room without the presence of others. Administration of questionnaires was made in the same order for all participants, and only PwMS completed the BICAMS.

The study was conducted in accordance with the Declaration of Helsinki ethical principles. The ethics committee of the University of Ioannina approved the study protocol (Reference number 15621/23-03-2022). All participants were informed of the nature of the study and provided written consent for their participation. Participants were notified they could opt-out of the study at any time as they wished, without any impact on their medical treatment (World Medical Association Declaration of Helsinki, 2013).

### Statistical analysis

Normality assumptions for the distribution of the CLAMS total score for both PwMS and HCs were evaluated using the Kolmogorov–Smirnov test, as well as QQ plots. The Independent samples t-test was used to evaluate the differences between the two groups on the CLAMS total score. Construct validity was evaluated using the Pearson correlation coefficient to explore the relationship between the CLAMS and age, as well as with the independent samples t-test to explore differences based on gender. Convergent validity was evaluated by correlating the CLAMS scores with the SMS, BDI-FS, and SAQOL-39 scores. Internal consistency was evaluated using Cronbach’s alpha. The estimated values were consistently high for the total sample, as well as for the two groups. Floor and ceiling effects were evaluated using the 15% of participants cut-off score suggested by Terwee et al. (2007). The statistical significance was set at α = 0.05 in all cases. SPSS (Statistical Package for the Social Sciences) 26.0 was used to carry out all statistical analyses.

## Results

### Study population

The study sample consisted of 106 PwMS (69.8% female) and 51 HCs (58.8% female). The mean age was 42.40 (±11.31) for PwMS and 43.24 years (±12.13) for HCs. The mean years of education was 13.5 (±3.00) for PwMS and 14.14 (±2.76) for HCs. There was no difference regarding age, sex, education level, disease duration, EDSS score, or MS type between PwMS and HCs (*p* > 0.05). The participants’ characteristics are presented in Table 1.

**Table 1:** Participants’ characteristics (sex, age, education years, EDSS score, time since diagnosis, and MS type)

	Male (*N* = 32)		Female (*N* = 74)			
PwMS	*M*	*SD*	*M*	*SD*	*t*(104)	*p*
Years of education	13.53	2.81	13.49	3.06	0.071	0.944
Age	42.69	10.61	42.27	11.67	0.174	0.863
EDSS score	2.13	1.22	2.26	1.35	0.479	0.633
Time since diagnosis	13.78	8.03	12.45	7.34	0.836	0.405
MS type PPMS/RRMS/SPMS (*N*, %)	2/28/2 (6.25% / 87.5% / 6.25%)		2/65/7 (2.7% / 87.7% / 9.5%)			0.765
	Male (*N* = 21)		Female (*N* = 30)			
HCs	*M*	*SD*	*M*	*SD*	*t*(49)	*p*
Years of education	14.71	1.82	13.73	3.23	1.258	0.214
Age	44.52	9.52	42.33	13.75	0.631	0.531

*N* = number; PwMS = Persons with Multiple Sclerosis; EDSS = Expanded Disability Status Scale; MS = multiple sclerosis; PPMS = primary progressive multiple sclerosis; RRMS = relapsing–remitting multiple sclerosis; SPMS = secondary progressive multiple sclerosis; HCs = healthy control group; *M* = mean; SD = standard deviation

### Discriminant validity scores and comparison between PwMS and HCs for the CLAMS total score

The mean score for the CLAMS total score was 18.13 (±6.63) for PwMS and 16.18 (± 4.14) for HCs. A statistically significant difference was observed between the two groups as shown in Table 2.

**Table 2:** Mean score and standard deviation for CLAMS questionnaire (PwMS and HCs)

	PwMS (*N* = 106)		HCs (*N* = 51)			
	*M*	*SD*	*M*	*SD*	*t*(155)	*p*
CLAMS	18.13	6.63	16.18	4.14	2.256	0.026

PwMS = Persons with Multiple Sclerosis; HCs = healthy control group; *N* = number; M = mean; SD = standard deviation; CLAMS = Communication and Language Assessment Questionnaire for persons with Multiple Sclerosis

### Convergent validity: Correlation between scores on the CLAMS and the SMS, SAQOL-39, and BDI-FS, and between the CLAMS and BICAMS

Pearson’s correlation between the CLAMS total score and SMS (*r* = 0.745; *p* < 0.001), SAQOL-39 (*r* = 0.682; *p* < 0.001), and BDI-FS (*r* = 0.450; *p* < 0.001) revealed strong positive significant correlations indicating that higher CLAMS scores are expected for higher SMS, SAQOL-39, and BDI-FS scores.

In addition, there was a statistically significant negative correlation between the CLAMS and the three subtests of the BICAMS (more specifically: SDMT [Rho = −0.274; *p* = 0.005], GVLT-II [Rho = −0.311; *p* = 0.001], and BVMT-R [Rho = −0.267; *p* = 0.006]).

### Test for internal consistency reliability coefficients for the CLAMS in PwMS, HCs, and the total sample

The internal consistency of all items was excellent. Cronbach’s alpha was 0.933 for PwMS and 0.851 for HCs. The estimates of the total sample were also high, equal to 0.921. This closely compares with the internal consistency of PwMS in the original CLAMS questionnaire; α = 0.944.

### Construct Validity

The CLAMS questionnaire showed no differences with regards to gender or age in the HCs, although a statistically significant weak positive correlation was observed in the PwMS group. A statistically significant negative, but weak, correlation was observed between education years and CLAMS score (*r* = −0.219; *p* = 0.006) indicating that lower CLAMS scores are expected for more years of education. The correlation between the CLAMS and disease duration (Rho = 0.023; *p* = 0.817), age (Rho = 0.105; *p* = 0.285), and EDSS scores (Rho = 0.118; *p* = 0.232) was not statistically significant. Finally, no differences were observed based on MS subtype as shown in the following table (Table 3) (*p* = 0.238) based on the independent samples *t*-test.

**Table 3:** CLAMS score in different MS subtypes

	CLAMS				
	*M*	*SD*	*N*	*F*(2,102)	*p*
PPMS	15.75	5.50	4	1.46	0.238
RRMS	17.87	6.40	93		
SPMS	21.44	9.07	9		
Total	18.10	6.65	106		

CLAMS = Communication and Language Assessment questionnaire for persons with Multiple Sclerosis; PPMS = primary progressive multiple sclerosis; RRMS = relapsing remitting multiple sclerosis; SPMS = secondary progressive multiple sclerosis; *M* = mean; SD = standard deviation; *N* = number; *p* = *p*-value

### Floor and ceiling effects

Amongst participants, 14.65% (15.1% PwMS and 13.7% HCs) had a CLAMS total score of 11, indicating low floor effects when using the >15% cut-off for PwMS. This is not significantly different to floor effects observed in the HCs. None of the PwMS had the maximum score; hence no ceiling effects were present in either group.

## Discussion

Our study presents the Greek version of the CLAMS, which is a PROM that evaluates communication symptoms in PwMS. The 11-items of the CLAMS address various communciation domains, including word finding difficulty, non-specific vocabulary, memory deficits in the verbal modality, insufficient information at discourse level, distractibility in conversation, linguistic non-fluency, and poor discourse structure. The internal consistency reliability coefficient of the Greek version of the CLAMS (*a* = 0.933 for PwMS) agrees with the original version (El-Wahsh et al., 2020b).There was a strong positive correlation between the CLAMS and the other PROMs (SMS, BDI-FS, and SAQOL-39). This finding is consistent with the literature, which supports that limitations in communication function and participation can be attributed to changes in speech and language (as evident in the SMS), to deficits in cognition, vision, mobility, and a susceptibility to fatigue (as evident in the SAQOL-39), as well as to emotional changes (as evident in the BDI; Carotenuto et al., 2018; Johansson et al., 2021; Yorkston et al., 2001). The strong negative correlation of the CLAMS with all three subscales of the BICAMS (SDMT, GVLT-II, and BVMT-R) agrees with other studies, which suggest that cognitive deficits affect 40%–65% of PwMS (De Looze et al., 2019) and can correlate with communication symptoms in MS (Halstead et al., 2021; Johansson et al., 2021). On the other hand, EDSS scores did not correlate with the CLAMS. The lack of emphasis on cognitive and communicative functions in the EDSS (Demir, 2022) might account for this result. Finally, there was no correlation between the CLAMS and participants’ age, disease duration, and MS type. Hence, communication-related deficits can be observed among any PwMS irrespective of such demographic factors, which concurs with other research (De Biagi et al., 2020).

As the prevalence of MS is increasing worldwide, the prevalence of MS in Greece has also substantially increased within a decade (Bakirtzis et al., 2020). According to the Atlas of MS (2013), the prevalence of MS in Greece is between 60 and 100 per 100,000 persons. More specifically, 21,218 (13,994 or 65.8% females, sex ratio 1.93:1) individual cases of MS have been identified in Greece. Further, there is growing recognition that the complex pathophysiology of MS can extend beyond medical and physical symptoms, and can negatively affect a person’s communication (Thompson et al., 2022). Consequently, structured screening, questioning, and reporting of communication symptoms is important to guide rehabilitation based on individual and specific needs (Tulek et al., 2017). Taking the earlier into account (i.e., the high proportion of PwMS in Greece and potential communication symptoms in MS) and the importance of ensuring PROMs are culturally and linguistically adapted (Gjersing et al., 2010), it was important and timely to translate and validate the CLAMS questionnaire into the Greek language. The results of the Greek version of the CLAMS show that it is a valid tool, which can be beneficial and used for various clinical and research purposes.

### Limitations

It is important to acknowledge the limitations of this study. Firstly, symptoms were evaluated only using PROMs (SMS, SAQOL-39, and BDI-FS) and no instrumental evaluation, such as acoustic voice analyses or formal communication batteries, were used to confirm patient-reported symptoms. In addition, the number of participants with progressive MS types were relatively small, hence it was not possible to compare the differences between MS subgroups. This can be a consideration for future research, as individuals with moderate or severe speech disorders are more likely to exhibit a progressive rather than a relapsing remitting type of MS (Yorkston et al., 2003), and thus communication changes may be even more prevalent. Nevertheless, the primary aim of this study was to adapt and validate the CLAMS questionnaire into the Greek language, and to ensure that results were applicable to most PwMS. Future research can be conducted with a larger sample, and to confirm the findings using instrumental communication analyses.

## Conclusion

Communication changes can have a major impact on participation and quality of life for PwMS, as well as on the efficiency of rehabilitation (Renauld et al., 2016). These symptoms are often overlooked and go undetected (El-Wahsh et al., 2022). The CLAMS can fill in this gap resulting from the lack of tools for assessing communication changes in MS. The results of this study indicate that the Greek version of the CLAMS has sound psychometric properties and satisfactory validity and reliability measurement properties. Accordingly, this tool may be used as a valid questionnaire to evaluate communication changes in PwMS in both clinical and research settings.

## Funding

No funding was received.

## Conflict of Interest

None declared*.*

## Author Contributions

Nefeli Dimitriou (Data curation, Investigation, Resources, Validation, Writing—original draft), Grigorios Nasios (Writing—original draft, Writing—review & editing), Anastasia Nousia (Resources, Visualization, Writing—original draft, Writing—review & editing) Emmanouil Anyfantis (Data curation, Resources), Lambros Messinis (Methodology, Validation), Georgios Dimakopoulos (Formal analysis, Software) Sarah El-Wahsh (Methodology, Writing—review & editing), Christos Bakirtzis (Software, Validation, Writing—review & editing), Vasiliki Kostadima (Data curation, Validation, Writing—review & editing) Spiridon Konitsiotis (Supervision, Writing—review & editing).

## A. APPENDIX

**Table:**

	**Ποτέ ή σπάνια (1)**	**Μερικές φορές (2)**	**Συχνά (3)**	**Συνήθως ή πάντα (4)**
1. Δυσκολευτήκατε να σκεφτείτε τη συγκεκριμένη λέξη που θέλατε;				
2. Δυσκολευτήκατε να ακολουθήσετε τον ειρμό της σκέψης σας καθώς μιλούσατε;				
3. Χρησιμοποιήσατε πολλές αόριστες ή ασαφείς λέξεις όπως “κατάλαβες τι εννοώ” αντί για την σωστή λέξη;				
4. Δυσκολευτήκατε να θυμηθείτε πληροφορίες που ήταν στην άκρη της γλώσσας σας;				
5. Δυσκολευτήκατε να εκφράσετε με ακρίβεια αυτό που θέλατε να πείτε;				
6. Παραλείψατε σημαντικές λεπτομέρειες;				
7. Δυσκολευτήκατε να συγκεντρωθείτε αρκετά ώστε να καταλάβετε τι ειπώθηκε;				
8. Δυσκολευτήκατε να θυμηθείτε πρόσφατες συζητήσεις;				
9. Δυσκολευτήκατε να παρακολουθήσετε τις κυριότερες λεπτομέρειες συζητήσεων;				
10. Δυσκολευτήκατε να συνδέσετε ιδέες με ένα λογικό τρόπο;				
11. Διστάσατε, κάνατε παύση ή επαναληφθήκατε;				
